# Modeling Polymeric Drug Release: The Emerging Role of Machine Learning

**DOI:** 10.1002/wnan.70057

**Published:** 2026-03-02

**Authors:** Ryan N. Woodring, Kristy M. Ainslie

**Affiliations:** ^1^ Division of Pharmacoengineering & Molecular Pharmaceutics Eshelman School of Pharmacy, UNC Chapel Hill North Carolina USA; ^2^ Department of Biomedical Engineering NC State/UNC Chapel Hill North Carolina USA; ^3^ Department of Microbiology and Immunology School of Medicine, UNC Chapel Hill North Carolina USA

## Abstract

Polymeric drug formulations have significantly improved the safety, efficacy, and clinical impact of many therapies. A persistent challenge for formulation scientists, however, lies in accurately characterizing time‐dependent drug release. For decades, researchers have relied on mathematical and physical principles, with a focus on transport phenomena, to interpret release kinetics from various polymeric systems using mechanistic and empirical models. While these models provide a foundational understanding through equations relating diffusion, swelling, and erosion, they often depend on simplifying assumptions and are often limited to a retrospective analysis of in vitro data. Recent advances in artificial intelligence (AI) have since opened the door for a new frontier in modeling strategies. Specifically, machine learning (ML) is being used not only to characterize drug release but also predict it while unveiling key formulation parameters governing unique kinetic profiles. This approach can support faster and more efficient development of polymeric systems. In this review, we explore how traditional drug release models have set the stage for ML in drug delivery research. We discuss important trends across recent ML applications, including data compilation, processing, architecture selection, and performance metrics. This perspective aims to provide scientists with a practical roadmap of ML applications used in formulation development. By integrating these tools with established knowledge, researchers can advance the design and translation of the next generation of polymer‐based drug delivery systems.

This article is categorized under:

Therapeutic Approaches and Drug Discovery > Emerging Technologies

## Graphical abstract

A review of mechanistic, empirical, and machine learning (ML) approaches for modeling drug release from polymeric systems, highlighting how data‐driven methods uncover relationships between formulation parameters and release behavior to guide future drug delivery design.

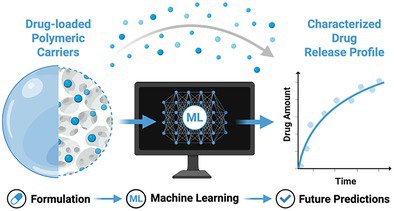

## 1 Introduction

Over the past 50 years, polymers have been evolutionary to the development of many life‐saving therapies. Through the design of polymeric formulations such as tablets, particles, and hydrogels, drug release can be tailored and controlled to optimize therapeutic outcomes (Sung and Kim 2020). In more recent years, polymeric systems have evolved from inert drug carriers to complex and stimuli‐responsive matrices while also improving the delivery of diverse therapies like small molecules, biologics, and even combination treatments (Woodring et al. 2023).

Since their adaptation into pharmaceutics, several polymers have achieved regulatory approval and clinical use (Alam et al. 2014). Biodegradable polyesters, namely poly(lactic‐co‐glycolic acid, PLGA), have been most widely adopted for offering tunability in degradation, and consequently, release. Others including polycaprolactone (PCL) and polyethers, like polyether polyethylene glycol (PEG), have also been explored in long‐acting formulations, each offering unique drug release profiles characteristic of the chosen material. Despite the advantages of polymers in drug delivery, critical evaluation of their dynamic features including degradation and drug release as carriers is an ongoing feat and pivotal to not only regulatory approval but also clinical efficacy and use (Gaspar and Duncan 2009).

For example, the carmustine (BCNU)‐loaded polyanhydride (prolifeprosan 20) wafer, Gliadel, was FDA approved in 1996 for the treatment of glioblastoma multiforme (GBM), but its clinical efficacy has been limited in part by an incomplete understanding of drug release from the wafer. This interstitial therapy is introduced at the time of GBM resection into the brain cavity to locally deliver the loaded chemotherapy (Iuchi et al. 2022; Perry et al. 2007). Poor clinical outcomes are likely because pre‐clinical studies lead to use of BCNU, a drug first identified as a chemotherapeutic in 1962, over paclitaxel (PTX), a more potent chemotherapeutic with first reported anti‐cancer activity in 1979 (DeVita et al. 1967; Wood et al. 1995). In the pre‐clinical scale up of these Polifeprosan 20 wafers from mice to non‐human primates (NHP), there was a substantial difference in release kinetics between the smaller mouse wafer compared to the larger NHP wafer. While Walter et al. reported 50% PTX release by 21 days in rodents, pharmacokinetic (PK) studies in NHPs with the larger wafer reported that less than 10% of PTX was released over 160 days (Walter et al. 1994). This is likely due to the decreased surface area to mass ratio with the larger wafer size since the polymer is surface‐eroding (Walter et al. 1994; Fung, n.d.).

Characterizing drug release from polymeric systems gives rise to several scientific and practical challenges. This concept is widely considered to be first illustrated in (Folkman and Long 1964). The authors developed a drug‐loaded silastic capsule to offer sustained and local release of cargo following interstitial delivery. This work inspired many others, paving the way for an emerging field in pharmaceutics; controlled drug delivery. Researchers like Dr. Robert Langer expanded upon Folkman's findings and characterized the release kinetics of proteins encapsulated in various polymeric matrices (Langer and Folkman 1976). This work highlighted how polymer, encapsulated cargo, and respective concentrations can influence observed release rates and inform the design of future polymeric formulations.

### 1.1 Traditional Mechanistic and Empirical Models for Drug Release

The mechanisms that govern release, such as diffusion, swelling, and degradation, often occur simultaneously in many polymeric platforms which can be easily influenced by polymer composition, molecular weight, porosity, and environmental conditions (Fu and Kao 2010; Liu and Quan 2016). Transport phenomena models of drug release from polymeric systems were first based on mechanistic models that incorporated fundamental physicochemical parameters like a drug diffusion coefficient and polymer degradation and erosion (Table 1). These models incorporated the earliest transport equations such as Fick's Law of Diffusion and the Navier‐Stoke equations—based on Euler's equation of continuity (i.e., mass balance) (Son et al. 2017). Series of partial differential equations were determined with boundary conditions applied to simplify these equations to fit the parameters experimentally determined.

**TABLE 1:** Traditional models for polymeric drug release.

Model	Description/basis	Strengths	Limitations
Mechanistic models			
General mechanistic models	Based on physicochemical principles like diffusion, degradation, erosion; includes Fick's Laws, Navier–Stokes, and continuity equations.	Well‐established Requires minimal data Interpretable and generalizable	Simplified assumptions Poor fit to complex or nonlinear systems
Diffusion‐controlled (Fick's laws)	Drug diffuses through polymer matrix; based on concentration gradients (Fick's First and Second Laws).	Simple models for steady and non‐steady state Strong theoretical basis	Assumes constant diffusivity Ignores swelling/degradation Sink conditions required
Higuchi model	Square‐root time release kinetics; assumes planar geometry, constant diffusivity, and uniform drug loading.	Easy to apply Good approximation in early‐stage systems	Not suitable for swelling or degrading systems
Swelling‐controlled (Peppas–Sahlin)	Models where swelling governs drug release; includes polymer relaxation and Fickian diffusion components.	Captures non‐Fickian behavior Suitable for hydrogels and hydrophilic polymers	Complex fitting Limited to specific polymer systems
Erosion‐controlled (the Hixson–Crowell cube root model)	Surface erosion–based release; applicable to systems where geometry remains constant and erosion dominates over diffusion.	Models zero‐order release Useful for surface‐eroding systems	Only fits surface‐eroding materials Geometry assumptions limit application
Combination (Siepmann, Göpferich)	Incorporate diffusion, swelling, degradation, and polymer relaxation; model biphasic/triphasic releases.	Captures complex behaviors More physiologically relevant	Requires computational/ML support complex parameterization
Empirical models			
Korsmeyer–Peppas	Semi‐empirical model; fits early release data with a release exponent to characterize Fickian versus anomalous transport.	Versatile Quick insight into mechanism Easy to apply	Limited to early‐stage release Geometry‐dependent No mechanistic insight
Numerical order (zero‐/first‐order)	Zero‐order: constant release rate first‐order: release rate proportional to remaining drug.	Intuitive Applicable to many systems	Oversimplified Poor fit to multi‐phase or nonlinear release
Weibull model	Empirical distribution function fitting sigmoidal, exponential, and parabolic profiles.	Very flexible Fits diverse datasets	No mechanistic interpretation Poor extrapolation
Logistic model	Empirical model capturing sigmoidal behavior (slow–fast–plateau phases).	Fits *S*‐shaped profiles well Useful for swelling/delay systems	Limited to sigmoidal patterns Not predictive
Polynomial models	Fits drug release using polynomial equations of varying degree.	Highly flexible Useful for interpolation	Risk of over fitting No mechanistic or predictive insight

Mechanistic models can be grouped as diffusion, swelling, erosion, combination and physicochemical dependent models (Tables 1 and S1) based on various mechanisms driving matrix degradation and drug dynamics. There are several strengths to these models that make them highly reliable for use in predicting drug behavior (Trucillo 2022). They primarily rely on foundational theories established centuries ago, making them reliable models for well‐characterized systems. Because of this historical context, the models are most often comprised of physicochemical parameters (e.g., diffusion co‐effects) which are well understood and can help predict release in simple new conditions based on modifications to the parameters. Further an additional appeal to mechanistic models is that they can be applied to sparse data systems. However, there are several limitations to mechanistic models, with the most significant being that they required mathematic simplifications to be functionally applied. These simplifications often cannot accurately model complex or variable environmental systems resulting in limited flexibility in the use of these models. Indeed, often mechanistic models cannot capture nonlinear or multi‐modal behavior, sometimes seen with polymer systems such as bulk‐degrading materials like PLGA and PCL (Ramchandani et al. 1997).

Whereas mechanistic models are based on fundamental physicochemical parameters, empirical models use a data‐driven curve‐fit that does not take into account the underlying mechanism of drug release from the polymer matrix (Table 1) (Koshari et al. 2019). The strengths of these models are that they are easy to apply and have a relatively low entry regarding coding or computational knowledge. They can be very useful in back‐of‐the‐envelope calculations to quickly approximate drug release and draw preliminary conclusions. However, this modeling approach also has limitations in that it often poorly extrapolates beyond the available data which leads to limited prediction and interpretation of the data. Because of this limitation, it is challenging to apply in formulation design or mechanistic understanding of the drug‐polymer system. These empirical models can fit data to common mathematical movements such as sigmoidal, exponential, or parabolic lines (Trucillo 2022). There are several empirical models that are commonly used to model drug release from drug‐polymer systems including the Korsmeyer‐Peppas, numerical order (e.g., zero‐order, first‐order), Weibull, logistic, and polynomial models.

Empirical models offer a practical and accessible means of analyzing drug release from polymer systems, especially when mechanistic understanding is limited or unavailable. Their strength lies in their simplicity and adaptability to a wide range of release profiles, making them valuable tools for early‐stage formulation screening and exploratory data analysis. However, their reliance on curve‐fitting without mechanistic grounding limits their predictive power and applicability in formulation design. As such, while empirical models like Korsmeyer–Peppas, Weibull, logistic, and polynomial functions are indispensable for descriptive modeling, they should be complemented with mechanistic insights or more advanced modeling approaches when deeper understanding or extrapolation is required (Djuris et al. 2024).

### 1.2 Introduction to Machine Learning for Drug Release

Traditional evaluation methods rely heavily on in vitro testing and mathematical modeling, but these approaches are oftentimes limited when capturing nonlinear or mixed‐phase behaviors that emerge, especially as new polymers and platforms are developed (Trucillo 2022; Pourtalebi Jahromi et al. 2020). This complexity makes it difficult to isolate the contribution of individual factors and to predict how modifications to formulation or geometry will affect overall kinetics. As the field moves toward increasingly multifunctional and stimuli‐responsive systems, there is a growing need for analytical and computational methods that can interpret data and reveal inter‐parametric relationships discerning unique release profiles. Recent advances in modern technology have introduced new tools that can overcome many of these challenges encountered with traditional and empirical strategies for characterizing drug release. The emergence of artificial intelligence (AI) and machine learning (ML) has provided researchers with a data‐driven framework for uncovering these trends learned directly from experimental observations (Aghajanpour et al. 2025; Protopapa et al. 2025a). ML applications have been successful at identifying nonlinear or multi‐variable dependencies within polymeric formulations, while also predicting a desired outcome with high precision and outperforming many traditional characterization methods (McDonald et al. 2023).

Many early ML applications in context to drug development involved the construction of artificial neural networks (ANNs), a computational algorithm resembling biological neurons and operating as nonlinear computational systems that combine weighted inputs at their synapses with nonlinear activation functions to produce an output (Rafienia et al. 2010). From quantitative‐structure activity relationship (QSAR) research to predicting patient outcomes, ANNs have been explored widely across the drug development pipeline demonstrating a vast range of suitable applications to determine non‐obvious relationships between inputs and outputs (Niculescu 2003; Bottaci et al. 1997).

Lending to their vast capabilities, ANNs have also been amended to aid researchers in the optimization of polymeric drug release formulations and characterize complex drug release kinetics (Vigata et al. 2020; Jesney et al. 2025). These early applications were explored primarily with drug releasing tablets, in which ANN constructs were commonly developed for capturing nonlinear, multivariate relationships across formulations through the hidden nodes and layers (Barmpalexis et al. 2010; Sun et al. 2003). Although reports commonly demonstrate overall good performance for tablet systems, many modern applications for more diverse platforms deviate from ANN constructs. This can be due to several challenges faced with ANN algorithms including a high reliance on large amounts of data, susceptibility to over fitting when fed “noisy” data, and poor generalizability across different or unique formulations (Zhang, Wang, et al. 2025; Ibrić et al. 2012). Additionally, many modern‐day polymeric formulations lack robust datasets or diverse formulation parameters suitable for ANN interpretation, leading to poor performance for making new predictions. Furthermore, ANNs require well‐optimized feature selection, scaling, and categorical encoding as their hidden layers are sensitive to differences in input magnitude and representation (Hassan and Baghban 2025). Without appropriate preprocessing, ANNs can learn unstable patterns and perform poorly when predicting new formulations.

Despite the power of ML capabilities, the success of computational tools like ML and AI to uncover trends in data for polymeric systems is often limited by the lack of comprehensive or extensive data available for algorithms to learn from (Meyer et al. 2023; Goren et al. 2025). As a result, many researchers have turned to compiling data reported across the literature; however, this necessitates consistent documentation of data from lab to lab for robust data curation (Goren et al. 2025). Furthermore, there is a lack in consistent ML findings pertaining to data design for ML documented in the literature which can be used for streamlining future data‐driven applications. As polymeric drug releasing formulations vary widely with respect to platforms, polymers, and drugs it is critical for researchers to define relevant parameters influential to ML predictions and mechanistic rationale for ML interpretations.

Another major challenge in the application of ML to polymer‐based drug delivery systems is the limited interpretability of many models, which can make it difficult for researchers to understand why a prediction was made or how specific features contribute to release behaviors. This lack of transparency is a significant concern in a field that relies on mechanistic understanding of material properties and release processes to inform future design, and it often leads to hesitation in adopting ML driven insights. To address this issue, researchers have begun using explanation tools such as SHapley Additive exPlanations (SHAP) assessment and Local Interpretable Model Explanations (LIME) that can reveal how individual parameters influence a model's output. Furthermore, incorporation of traditional mechanistic models within ML frameworks, such as the use of ML to predict rate constants involved in mathematical equations, has demonstrated success for linking data‐driven predictions with established release mechanisms. Together, these approaches improve confidence in ML predictions and support deeper mechanistic insight, making ML more useful for the rational design of polymeric formulations.

Overall, harnessing of ML tools and capabilities has been most successful for characterizing polymeric drug release when (1) robust and appropriately preprocessed data is made available; (2) built from well‐defined features relevant to the system, formulation, and drug release mechanisms; and (3) when interpretation tools are assessed to validate ML predictions. Common methods for adopting ML strategies have been explored with several reviews detailing how these tools can be implemented for diverse formulation systems (Aghajanpour et al. 2025; Meyer et al. 2023; Gormley 2024; Jena et al. 2024). Despite the growing knowledge and current information available on ML frameworks, there lacks a broader consensus when it comes to compiling drug release datasets based on ML findings across the field. In this review, we have outlined a structural roadmap for curating optimized polymeric drug release datasets which can enable ML technology by navigating trends seen across recent applications. We also provide a consensus on the critical considerations, relevant interpretations, and broader implications for these data‐driven strategies explored across this rapidly growing field.

## 2 Machine Learning Workflows for Polymeric Drug Release

A typical ML workflow begins with the collection and curation of relevant experimental data, followed by preprocessing steps such as normalization, feature selection, and encoding (Vamathevan et al. 2019). Once the dataset is prepared, algorithms are trained to learn relationships between input features such as polymer, drug, and formulation parameters, and a defined output, such as cumulative release or rate constants (Bannigan et al. 2021). Model performance is then evaluated using statistical metrics and cross‐validation measures to ensure predictive accuracy and generalizability is achieved before interpretation or applications with new formulations (Wilson et al. 2025). The following sections provide an overview of common ML concepts for characterizing drug release.

### 2.1 Supervised, Unsupervised, and Deep Learning Approaches

ML encompasses a range of learning paradigms, each suited to different types of problems encountered in drug release modeling from polymer systems (Table 2) (Hathout 2021). Supervised learning is where models are trained on labeled datasets to predict known outputs and it is commonly used for regression tasks such as predicting drug release profiles based on formulation variables (e.g., polymer type, concentration, and drug loading) (Morales and Escalante 2022). Unsupervised learning, which uncovers hidden patterns without predefined labels, can be applied to cluster formulations with similar release behaviors or detect anomalies in experimental data (Trezza et al. 2024). Semi‐supervised learning leverages small amounts of labeled data alongside large unlabeled datasets, which is useful when experimental data is scarce or costly, as often seen in bioinformatics or initial screening studies (Reddy et al. 2018). Reinforcement learning, though less common in drug delivery, has potential in optimizing formulation strategies through iterative trial‐and‐error (Jayaraman et al. 2024). Deep learning, a subfield of ML focused on modeling complex, nonlinear relationships, is particularly well‐suited for learning from large, high‐dimensional datasets, such as those describing time‐dependent drug release kinetics (Husseini et al. 2025). Finally, self‐supervised learning, which has enabled breakthroughs in transformer‐based architectures and large language models, holds promise for pre‐training models on broad formulation datasets before fine‐tuning on specific drug systems (Zhao et al. 2024). Together, these ML paradigms offer powerful, flexible tools for understanding and optimizing drug release from polymer‐based delivery systems.

**TABLE 2:** Machine learning paradigms and applications.

Type	Key goal	Common use cases
Supervised	Predict known outputs	Classification, regression
Unsupervised	Discover patterns or structure	Clustering, anomaly detection
Semi‐supervised	Improve learning with few labels	Natural language processing, bioinformatics
Reinforcement	Learn from environment	Games, robotics, control systems
Deep learning	Learn complex representations	Vision, language, audio
Self‐supervised	Pre‐training or feature learning	Transformers, large language models

### 2.2 ML Algorithms

**FIGURE 1:**
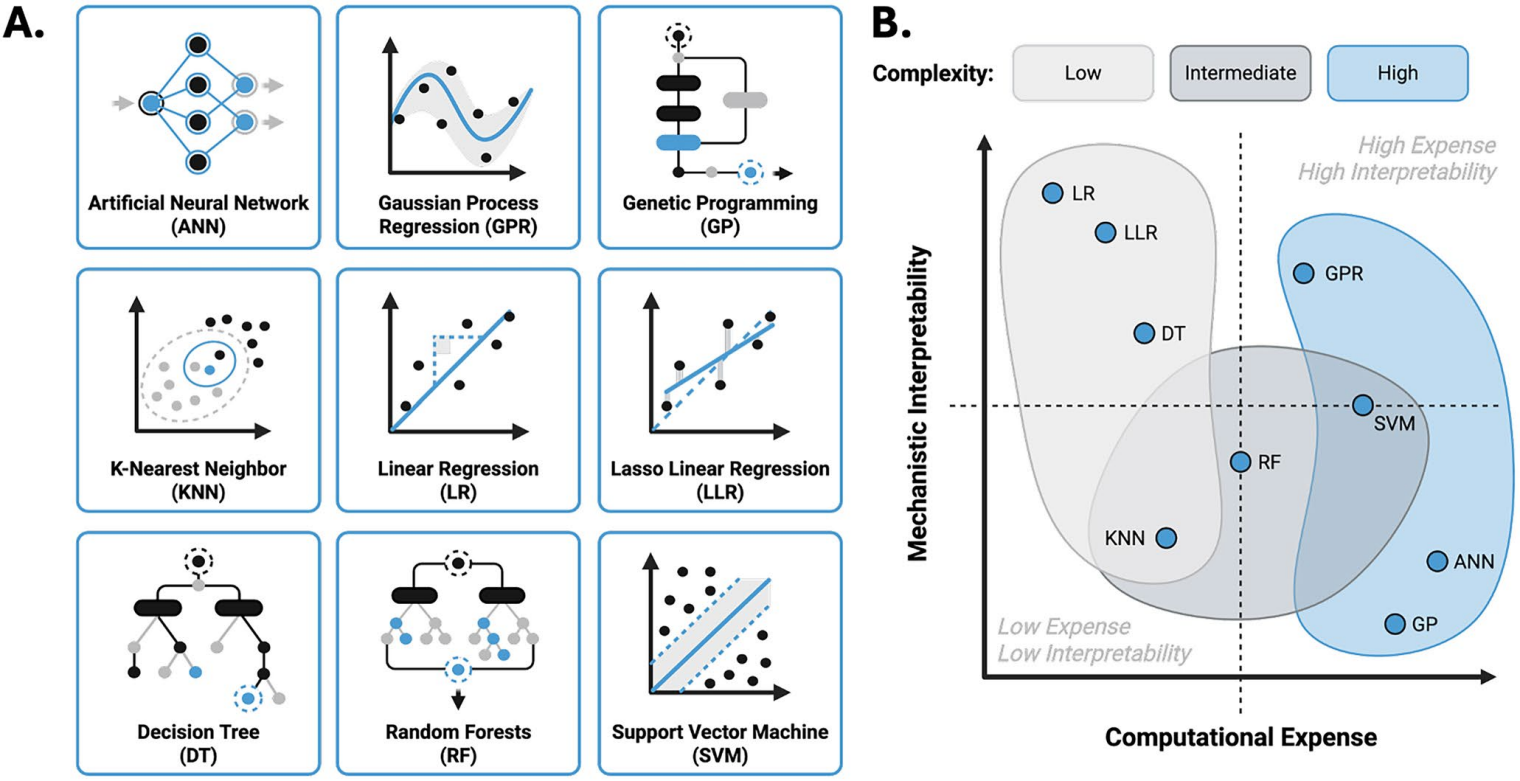
Common machine learning architectures. (A) Nine commonly used ML algorithms for drug release with depicted architecture. (B) Distribution of ML models with respect to computational expense (*x*‐axis), mechanistic interpretability (*y*‐axis), and grouped in shaded regions by overall complexity. Overlapping regions represent models with variable complexity based on various ML factors.

These ML paradigms have been applied using common ML algorithms to model drug release, including linear (LR) and lasso linear (LLR) regression, ANNs, support vector machines (SVMs), decision trees (DT), random forests (RF), Gaussian process regression (GPR), *K*‐nearest neighbors (KNN), and Genetic Programing (GP) (Figure 1A). For applications of ML in the context of drug delivery, researchers should consider overall complexity, computational expense, and mechanistic interpretability of such models to ensure that drug release predictions are robust yet generalizable (Figure 1B).

ML models used to predict polymeric drug release often span probabilistic, kernel‐based, and neural approaches, each offering distinct strengths depending on data size and formulation complexity. GPR and SVM, for example, are well‐suited for situations where datasets are small (< 1000 release observations) and relationships between polymer properties and drug release are highly nonlinear (Woodring et al. 2025a; Cervantes et al. 2020). GPR can provide smooth predictions and uncertainty estimates that are useful for interpreting time‐dependent release profiles, while SVMs handle high‐dimensional inputs with strong resistance to over fitting (Kirk and Stumpf 2009).

ANNs have extended beyond initial applications while maintaining their capability of learning complex relationships among multiple formulation variables, demonstrating to be especially powerful for systems involving swelling, degradation, or multiphasic release. When applied to cumulative release versus time data, recurrent neural networks, for example, can capture temporal patterns more effectively than static models. However, most ANN‐based methods still require large, high‐quality datasets and often suffer from limited interpretability (Visan and Negut 2024).

A second group of widely used frameworks includes DTs, RFs, and boosted ensembles, which excel at modeling nonlinear and interacting formulation factors (Shi et al. 2019; Sousa et al. 2025). DTs provide transparent if‐then rules that can reflect mechanistic aspects of release, while RFs and boosted trees improve accuracy by combining many diverse learners. These ensemble models often highlight which variables have the strongest influence on release rates, such as polymer molecular weight, drug loading, excipient content, or environmental conditions. KNNs share similar features to this group of data‐driven algorithms. It predicts drug release behavior by referencing the most similar formulations in a dataset and is easy to implement when features are well‐scaled and curated (Obaido et al. 2024).

Several ML models are also commonly favored for their high interpretability: LRs, LLRs, and GPs. LRs and LLRs offer simplicity and help identify dominant formulation variables by shrinking unimportant features, making them useful for preliminary screening or systems with modest complexity (Shi et al. 2019). GP automatically evolves mathematical expressions that describe release profiles and can reveal mechanistic insights when the release pathway is uncertain or influenced by nonlinear interactions (Barmpalexis et al. 2011). Together, these ML methods demonstrate the versatility of data‐driven tools for understanding and predicting drug release across diverse polymer systems, offering advantages over traditional mechanistic and empirical models by enabling faster optimization, capturing complex behavior, and supporting deeper mechanistic insight.

Moreover, the wide range of ML algorithms used to model drug release from polymer systems each present distinct strengths and limitations (summarized in Table 3), which can make choosing the most appropriate method challenging. Aghajanpour et al. provided a more comprehensive review of these models, and several other studies offer guidance on how to appropriately construct ML architectures for drug release (as implementation details are beyond the scope of this review) (Aghajanpour et al. 2025; Meyer et al. 2023). In summary, these algorithms provide a diverse toolbox for accurately modeling drug release, with appropriate selection driven by data size, complexity, and the desired balance between interpretability and predictive power.

**TABLE 3:** Strengths and limitations of machine learning constructs.

ML method	Strengths	Limitations
Artificial neural networks (ANN)	Models nonlinear, high‐dimensional relationships Useful in complex, poorly understood systems	Requires large, high‐quality datasets Low interpretability “black box”
Decision trees (DT)	Simple and interpretable Works with small datasets No feature scaling needed	Prone to over fitting Unstable with data changes Lower accuracy alone
Gaussian process regression (GPR)	Ideal for small datasets Captures uncertainty via confidence intervals	Computationally expensive Requires careful tuning and may overfit
Genetic programming (GP)	Produces interpretable, symbolic models Flexible for unknown or nonlinear systems	Computationally intensive Prone to over fitting without proper control
Linear regression (LR)	Simple, interpretable model Fast and efficient on small datasets	Assumes linearity Poor performance with complex or nonlinear data
Lasso linear regression (LLR)	Performs feature selection Helps prevent over fitting Useful with high‐dimensional data	Can eliminate useful features May underperform with correlated inputs
*K*‐nearest neighbors (KNN)	Intuitive and easy to implement No training phase required	Slow with large datasets Sensitive to noisy, unscaled, or irrelevant features
Support vector machines (SVM)	Works well with small, complex datasets Captures nonlinear patterns Robust to over fitting	Computationally intensive for large data Sensitive to noise and parameter tuning
Random forests (RF)	Improves accuracy and stability over DTs Ranks feature importance	More computationally demanding than DTs May perform poorly with imbalanced data

### 2.3 Metrics for Model Comparison

When evaluating predictive models, there are several key metrics that are commonly used and the choice of these metrics depends on many important factors. Sensitivity to outliers also plays a role, as some metrics are more robust to extreme values than others. Interpretability needs to influence the selection as well; for instance, percentage‐based errors may be preferred in some contexts, while absolute errors may be more meaningful in others. Additionally, domain‐specific concerns must be considered, such as whether over‐predictions carry more risk or consequences than under‐predictions, which can significantly impact the prioritization of certain metrics (Rashidi et al. 2023). Common metrics (summarized in Table 4) are root Mean squared error (RMSE), mean absolute error (MAE), coefficient of determination (*R* ^2^), mean squared error (MSE), mean absolute percentage error (MAPE), cross‐validation scores, learning curve analysis, Akaike information criterion (AIC), Bayesian information criterion (BIC), and uncertainty quantification (UQ) (Miller et al. 2024; Emiliano et al. 2014).

**TABLE 4:** Common performance metrics for machine learning.

Metric	Description	Interpretation	Notes
Root mean squared error (RMSE)	Square‐root of the average squared prediction errors	Lower RMSE = better accuracy	Penalizes larger errors more strongly
Mean absolute error (MAE)	Average of absolute differences between actual and predicted values	Lower MAE = better performance	Less sensitive to outliers than RMSE
*R*‐squared (*R* ^2^)	Proportion of variance in the target explained by the model	Closer to 1 = better model fit	Can be negative if model performs worse than the mean
Mean squared error (MSE)	Average of squared differences between predicted and actual values	Lower MSE = better accuracy	Similar to RMSE but without square‐root
Mean absolute percentage error (MAPE)	Average of absolute percentage differences	Lower MAPE (%) = more accurate predictions	Not defined when actual values are zero
Cross‐validation scores	Performance measured across multiple training/validation splits	Higher consistency = better generalization	Helps detect over fitting or under fitting
Learning curve analysis	Plots of training versus validation errors over different training set sizes	Shape indicates over fitting or under fitting	Diagnostic rather than a single value metric
Akaike information criterion (AIC)	Measure of model quality balancing fit and complexity	Lower AIC = better model (penalizes complexity)	Useful for model selection, especially in regression
Bayesian information criterion (BIC)	Similar to AIC but with stronger penalty for complexity	Lower BIC = better model	Favors simpler models more than AIC
Uncertainty quantification (UQ)	Compares variance of inputs, model structure, and predictions to quantify confidence	Low UQ = within variance High UQ = outside variance	Assesses model reliability, suitable for extrapolation

Error‐based metrics such as RMSE, MAE, MSE, and MAPE directly measure discrepancies between predicted and observed release values, with lower values indicating stronger performance. These metrics differ in how they penalize error magnitude, with RMSE emphasizing large deviations and MAE offering a more outlier‐resistant assessment. RMSE is particularly favored for evaluating model performance because it is expressed in the same units as the predicted outcome (such as percent drug release or fractional release), making its magnitude easy to interpret. Goodness‐of‐fit measures such as *R* ^2^ complement these by indicating how much of the variance in release behavior is explained by the model, especially reliable for linear models but not always for more complex constructs (Chicco et al. 2021). Beyond point‐estimate accuracy, model selection criteria such as AIC and BIC evaluate performance while accounting for model complexity, supporting balanced comparisons across architectures; especially useful for comparison across ML and/or classical models (Stiepel et al. 2022). Together, these metrics help determine whether a model captures the underlying release behavior, over fits the dataset, or requires simplification or retraining.

Generalization and diagnostic tools such as cross‐validation scores and learning‐curve analysis provide additional insight into model reliability across different data distributions and training set sizes (Deng et al. 2023). Cross‐validation identifies inconsistencies that suggest over fitting or under fitting, while learning curves reveal whether improvements depend on more training data or better model design. Other assessment strategies, including UQ, further enhance evaluation by estimating how confident a model is in its predictions. UQ methods assess how variation in inputs or model structure affects predictive confidence and are especially valuable for polymer‐drug systems with sparse or heterogeneous datasets (Nemani et al. 2023; Woodring et al. 2025b). In this context, approaches such as GPR provide intrinsic confidence intervals, enabling researchers to identify regions of strong support versus areas of high uncertainty. Incorporating UQ in model evaluation promotes responsible interpretation of ML outputs and strengthens efforts to optimize formulation design and advance the translational potential of polymeric drug delivery systems (Tang et al. 2025).

### 2.4 ML Interpretation Tools

Interpreting machine learning models is crucial for advancing drug release predictions from polymeric systems. SHAP analysis is increasingly used in ML to interpret complex models by quantifying the contribution of each input feature to individual predictions (Prendin et al. 2023). In the context of predicting drug release from polymeric systems, SHAP helps identify which formulation parameters, such as polymer molecular weight, drug loading, or degradation rate, most strongly influence the release profile, which can be used to simplify model inputs. Similarly, LIME offers a complementary approach by generating simple local surrogate models that explain how small changes in inputs affect the prediction for a single formulation (Hassan et al. 2025). Together, SHAP and LIME improve transparency and increase confidence in ML‐derived insights by linking model outputs to meaningful material and drug characteristics.

Another valuable tool is agglomerative hierarchical clustering analysis, which enables researchers to group similar drug release profiles or formulations based on shared characteristics, uncovering underlying patterns in the data without needing labeled outcomes (Zhang et al. 2017). This clustering approach can guide formulation categorization and reveal structure within complex datasets and understand what design parameters most influence drug release. Altogether, these interpretive methods and evaluation metrics provide powerful means for understanding, comparing, and refining mechanistic, empirical, and ML‐based models in drug delivery research.

## 3 Defining the Data for ML: Platforms, Polymers, Drugs

**FIGURE 2:**
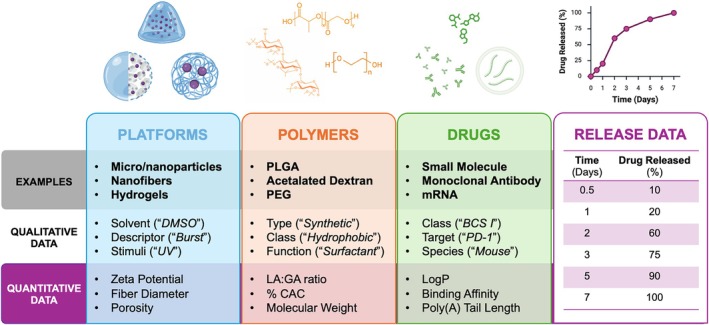
Dataset features for ML with polymeric drug release. Visual depictions of various platforms, polymers, drugs, and release data shown respectively. Bulleted lists include examples for described systems and various qualitative and quantitative measures used in datasets. Text in (“”) indicates a specific representation for the respective qualitative data.

A critical first step to harnessing ML capabilities is consolidating drug release data and defining relevant parameters associated with these observations and to be interpreted by ML for predicting the desired outcome (Yang, Liu, et al. 2024). When considering all that goes into the design of polymeric drug delivery systems, namely the polymers and drugs, ML applications can explore a wide range of qualitative and quantitative parameters associated with formulated platforms and corresponding drug release data (Figure 2). By incorporating multiple, varied, and interdependent features, ML models can capture both continuous and nonlinear relationships built from these features and provide accurate predictions of drug release. The qualitative and quantitative descriptors serve as the input parameters for ML frameworks, making their identification and optimization critical to model performance. Studies have varied in the number of input parameters from as few as 4 features to over 1500 (Jiang et al. 2025; Abdalla et al. 2024). Collectively, these parameters can not only enhance predictive accuracy across diverse systems but also provide mechanistic insights into the interplay between polymer chemistry, formulation design, and drug release kinetics, providing information that can guide rational design of next‐generation controlled release platforms.

### 3.1 Data Considerations and Preprocessing

Various ML applications differ not only by the platforms, polymers, and drugs being assessed, but also the breadth of unique features defined as input parameters for model interpretation (Woodring et al. 2025b; Abdalla et al. 2024; Owusu et al. 2025; Bannigan et al. 2023; Zhang, Zhang, et al. 2025a). Because these parameters originate from many sources (with respect to composition and characterization) and span different units, scales, and measurement standards, careful preprocessing is essential to harmonize the compiled data and ensure that models learn meaningful relationships rather than artifacts of inconsistent reporting (Maharana et al. 2022).

Common data preprocessing methods (listed in Table 5) involve data normalization strategies such as *z*‐score normalization, where each feature is transformed based on its mean and standard deviation, and min to max scaling, which rescales values within a defined range (e.g., 0–1) (Zelaya 2019). These techniques ensure that variables measured on different scales, such as polymer molecular weight (kDa) versus drug loading capacity (%wt.), contribute proportionally during model training and reduces bias toward features with larger numerical ranges, improving model convergence and interpretability. Outlier detection or removal is also important because extreme or inconsistent data points can distort learned relationships, reduce model stability, and lead to inaccurate release predictions that do not reflect true formulation behavior (Mohammed et al. 2025).

**TABLE 5:** Data preprocessing strategies.

Preprocessing method	Description	Applicable data types (examples)
Normalization (min–max scaling)	Rescales to a fixed range (0–1), preserves relative relationships while removing magnitude differences	Continuous variables (concentration, size, pH, temperature, polymer molecular weight, time)
Standardization (*Z*‐score scaling)	Transforms data mean = 0 with standard deviation = 1	Continuous variables with wide ranges (physicochemical properties and formulation parameters)
Log or power transform	Reduces skewness and stabilizes variance by applying log or power functions	Skewed features (solubility, particle size, degradation rates, release values)
One‐hot encoding	Converts categorical variables into binary columns for each category	Discrete identifiers (polymer type, drug class, processing method, release environment)
Label encoding	Assigns numeric labels to categorical classes using integer values	Ordered categorical data such as pH category (acidic, neutral, basic) or temperature category
Principal component analysis (PCA)	Reduces dimensionality by projecting data into smaller, latent components that capture the most variance	High‐dimensional continuous data (Raman spectra, NIR spectra)
Filtering/noise reduction	Removes high‐frequency noise or baseline drift with smoothing or filtering algorithms	Spectroscopic data (Raman, FTIR, UV–Vis), release profile time‐series, sensor‐based measurements.
Imputation (mean, median, mode)	Fills missing values using central tendency measures to maintain dataset integrity.	Experimental conditions, assay parameters, missing release values
*K*‐nearest neighbors imputation	Replaces missing values using most similar feature values	Mixed datasets with both categorical and numerical missing entries
Outlier detection and removal	Removes outliers using statistical rules or distance‐based methods	Numerical variables (release, time, polymer characteristics)
Data augmentation	Expands datasets with modified versions of existing samples or generating synthetic samples	Small datasets (limited time points, or formulation conditions)
Time‐series alignment or interpolation	Aligns irregularly sampled time points and interpolates missing values	Drug release profiles, degradation curves, sensor time‐signals

Time scaling is especially critical when compiling drug release data because release observations often include uneven sampling schedules, long experimental durations, or early burst release phases that dominate in unscaled data (Meyer et al. 2023; Desai et al. 2012). Scaling or transforming time allows models to recognize trends across studies that use different sampling frequencies or observation windows and prevents late time points from overshadowing early release features. For applications when data is compiled from various sources or describes complex features (e.g., spectroscopic or sensor‐based measures), methods for filtering noise are also encouraged since analytical measurements often contain experimental fluctuations, baseline drift, or instrument‐related artifacts that can distort meaningful interpretations (Meyer et al. 2023). Smoothing or denoising these signals allows ML models to focus on meaningful trends in the data, improving the reliability of predictions and reducing the risk of learning patterns that arise only from measurement noise rather than real formulation effects.

After preprocessing descriptive features and release data, researchers typically organize parameters into structured matrices where each formulation is represented by a set of polymer, drug, and experimental attributes along with any relevant release descriptors (Figure 2). Across the field, these compiled and processed datasets have developed a range of ML architectures with diverse predictive outcomes (Table 6). Altogether, these results have revealed interdependent relationships among common formulation parameters identified through these computational approaches. The following sections describe these patterns in ML driven drug release with respect to platforms, polymers, and drugs across various applications.

**TABLE 6:** ML applications for polymeric drug release.

Platform(s)	(*n*)	Polymer(s)	Drug(s)	Data size	Input parameters (*n*)	ML model	Error metrics	References
Compressed tablets	377	Hydrophilic, hydrophobic, amphiphilic excipients	Multiple BCS‐class APIs (20)	4147	Excipient type and %, API%, pH, time	Random forest (RF)	*R* ^2^, RMSE	(Protopapa et al. 2025b).
Coated pellets	13	Polysaccharides	5‐ASA	155	Raman spectral features (> 1500), media (3), time	RF	*R* ^2^, MSE, MAE	(Abdalla et al. 2024).
Aerogels	20	PUA and PIR‐PUR	5‐FU, paracetamol	80	*K*‐index, porosity, surface area, macro/mesopore ratio, pore radius, time	Linear regression (LR)	*R* ^2^, RMSE	(Owusu et al. 2025).
Ace‐DEX nanofibers	30	Ace‐DEX (varied %CAC)	Small molecules (8)	929	Drug MW, LogP, PSA, pKa; Ace‐DEX %CAC; drug load %; fiber diameter; time	Gaussian process regression (GPR)	*R* ^2^, RMSE, MAE, MSE	(Woodring et al. 2025b).
Long‐acting injectables	181	PLGA, PLA, PCL, PEA	Small molecules (21)	3783	Drug (7) and carrier features (7), time of release (3)	Tree‐based gradient boosting (LGBM)	AE, MAE	(Bannigan et al. 2023).
PLGA particles	43	PLGA, PVA, PLH, Lactel, PEG, Polyscitech, PAM	Multiple	97	pH, particle size, drug MW, solubility; time	GPR	MAE, RMSE, *R* ^2^	(Sun et al. 2025).
PLGA systems	39	PLGA (varied MW)	Multiple (33)	796	Carrier features (6), drug descriptors (14), time	Multilayer perceptron (MLP) neural network	*R* ^2^, MSE	(Zhang, Zhang, et al. 2025b).
Ace‐DEX microparticles	22	Ace‐DEX (varied %CAC)	Multiple (4)	924	Drug MW, LogP, PSA; Ace‐DEX %CAC; drug load %; pH; time	Neural network (NN)	MSE	(Stiepel et al. 2022).
PEDOT matrices	1	PEDOT	Ibuprofen	51	12 assay parameters: numerical (6); categorical (6)	RF	*R* ^2^, RMSE, MAPE	(López‐Flores et al. 2025).
HPMC tablets	168	HPMC	Drotaverine	8904	Tablet compression force; drug content; polymer content; particle size distribution; NIR spectra	Artificial neural network (ANN)	RMSE, *f*_2_	(Galata et al. 2021).
Chitosan nanoparticles	115	Chitosan	Small Molecules	194	Particle (2), formulation (2), polymer (3), drug (3), assay (2)	RF	*R* ^2^, RMSE	(Rastegari et al. 2025).
Microneedle patch	7	Plastic (Li et al. 2015) or PEGDA (Kochhar et al. 2012) (hydrogel)	Multiple (5)	191	Drug (MW, load); patch (type, length, SA); media; time	Extreme gradient boosting (XGBoost)	*R* ^2^, RMSE	(Yuan et al. 2023).

*Note:* Each report extracted from the cited work (references). Details central to the characterized platforms (*n* = total number of unique entries), polymers, drugs, parameters, best performing ML model construct, and assessment metrics.

Abbreviations: Ace‐DEX, acetalated dextran; HPMC, hydroxypropyl methylcellulose; Lactel, commercial medical‐grade PLGA (Lactel brand); PAM, polyacrylamide; PCL, polycaprolactone; PEA, poly(ester amide); PEDOT, poly(3,4‐ethylenedioxythiophene); PEG, polyethylene glycol; PEGDA, polyethylene glycol diacrylate; PIR PUR, polyurethane based polyisocyanurate; PLA, polylactic acid; PLGA, poly(lactic‐co‐glycolic acid); PLH, poly‐L‐histidine; Polyscitech, commercial PLGA or PLA polymers produced by PolySciTech; PUA, polyurethane acrylate; PVA, polyvinyl alcohol.

### 3.2 Polymeric Platforms

When defining platform‐specific parameters for machine learning models, researchers have drawn from a broad range of features that capture the structural and compositional diversity of polymeric drug‐delivery systems, including tablets, hydrogels, nanofibers, particles, and microneedles. These features typically encompass formulation components, such as polymer type, drug identity, excipients, surfactants, or binders, as well as experimentally measured characteristics like tablet compression force, fiber or particle diameter, porosity, and other physicochemical descriptors. Collectively, these parameters not only distinguish release behaviors across formulations but also enable ML models to learn patterns consistent with established principles in controlled drug delivery, providing a mechanistic foundation for predictive performance.

Various ML models have been developed solely on platform‐specific parameters, elucidating differences by characterized features across consistent or few polymer‐drug compositions. Owusu et al. for example, evaluated several ML models for predicting time‐sensitive drug release from polyurea (PUA) and poly(isocyanurate‐urethane) (PIR‐PUA), dextran, and dextran aldehyde‐coated silica aerogels; three‐dimensional, porous, nanostructured, drug delivery systems (Owusu et al. 2025). For the compiled drug release dataset and across 20 unique formulations, they defined 5 unique parameters as ML inputs (i.e., *K*‐index, porosity, surface area, macropore/mesopore, particle radius) along with time. These features describe unique and material‐specific differences among formulations represented as quantitative data. After training and testing various models with each dataset, the linear regression model achieved highest *R* ^2^ and lowest RMSE. SHAP assessment revealed that macropore/mesopore ratio, which describes the relative distribution of pore sizes, is the most influential feature for predicting drug release profiles. This was expected since they previously found that this feature of aerogels contributes stepwise to release (Bang et al. 2014).

To identify which parameters are most influential for prediction, researchers often perform feature selection or feature ranking methods before or alongside model training. Statistical *F*‐tests, for example, can be used to quantify a parameter's linear association to the desired response through comparisons of feature variance relative to residual variance (Gorriz et al. 2025). This necessitates that features are numerical, such as platform characterization measures.

**FIGURE 3:**
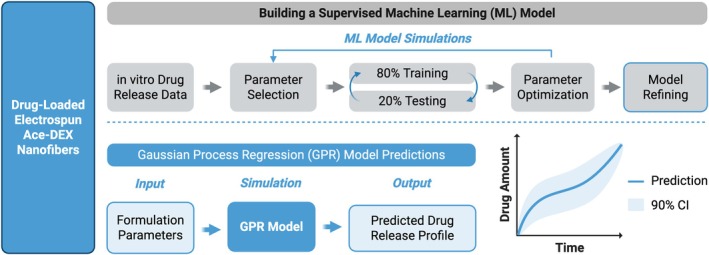
Supervised ML model for drug release from polymeric scaffolds. Parameters were first defined for each of the in vitro observations, followed by applying an 80:20 split to the data set. Parameters were optimized to reduce error of the training and testing simulations. Model parameters were then refined and evaluated for predictability. The final model interprets nanofiber formulation parameters and predicts drug release versus simulated time with a 90% confidence interval (CI, depicted in graph).

Woodring et al. applied this strategy throughout model development to optimize model performance based on the most influential features describing 30 unique electrospun acetalated dextran (Ace‐DEX) scaffolds; drug‐loaded, polymeric nanofibers with tunable degradation rates correlated to the relative cyclic acetal coverage (% CAC) along the Ace‐DEX polymer backbone (Figure 3) (Woodring et al. 2025a). These features were identified through iterative model simulations and performance assessments (*R* ^2^, MAE, MSE, and RMSE) associated to the parameters defined. Initially, drug‐specific (i.e., molecular weight, LogP, polar surface area, and pKa) and platform‐specific (i.e., Ace‐DEX % cyclic acetal coverage—%CAC, % drug wt. loading, fiber diameter) parameters were included in these simulations. Interestingly, all 4 scaffold‐specific parameters outranked drug‐specific parameters via statistical *F*‐tests and importance score rankings. When repeating the training and testing of ML models, the best performing ML model, a GPR construct, had optimum performance when only these top 4 scaffold features were defined as ML inputs for predicting drug release (*R* ^2^ = 0.931, MAE = 0.058, RMSE = 0.0843). This model using streamlined input parameters adequately predicted release profiles across several unique formulations, including a protein‐loaded system from a similar Ace‐DEX scaffold formulation (*R* ^2^ = 0.875). Altogether, these results highlight the importance of considering unique, yet distinct, input parameters when using ML to predict drug release across various formulations.

There is a consistent high influence seen for features describing platform surface morphology across ML applications for drug release (Luraghi et al. 2021). Yuan et al. similarly found this to be true upon developing an XGBoost model to predict release of drugs from microneedle patches into rat or human skin (Yuan et al. 2023). These drug‐loaded platforms comprised of both plastic and hydrogel (poly(ethylene glycol) diacrylate, PEGDA) materials with a range of cargo (small molecules, peptides, and proteins), and the dataset input parameters included skin type, 3 microneedle features (type, length, surface area), drug MW, drug loading, and permeation time. The model adequately captured drug % permeation across formulations (*R* ^2^ = 0.98), outperforming derivations using Fick's Law (*R* ^2^ = 0.82). Notably, microneedle surface area and permeation time were identified as highly influential features in the ML predictions, reinforcing the importance of considering these platform‐specific parameters for their mechanistic relevance to characterizing and predicting drug release.

### 3.3 Polymers

Across drug delivery platforms, polymers show broad variation in their chemistry, architecture, degradation behavior, and processing conditions. Because the polymer defines the bulk material, selecting meaningful input parameters is essential for capturing the factors that influence release (Patel and Webb 2024). Common parameters include physicochemical characteristics such as MW, hydrophobicity, and monomer ratios, which shape material stability and degradation kinetics. As the field of polymer science continues to advance, the number of unique and identifiable polymer features has expanded. Researchers now develop materials with dynamic responses to specific stimuli or environments, adding further complexity to the range of parameters that can influence drug release (López‐Flores et al. 2025). These defined features help ML models identify material‐based patterns that govern time‐dependent release across various matrices.

One of the most widely explored polymers in drug delivery is polylactic‐co‐glycolic acid (PLGA) (Kapoor et al. 2015). PLGA is a copolymer composed of lactic acid (LA) and glycolic acid (GA) monomers with distinct properties linked through ester bonds. Its degradation into naturally occurring metabolites, along with tunable mechanical and chemical properties, has made PLGA one of the most widely used materials in drug delivery (Yang, Zeng, et al. 2024). By adjusting the ratio of monomers, researchers can control polymer hydrophobicity, degradation rate, and cargo release profiles, enabling the design of particles, fibers, implants, and other platforms suited for controlled drug release. PLGA is also favored for its biocompatibility, its history of FDA approval in multiple products, and its ability to encapsulate a broad range of therapeutic agents. As a result, many researchers have explored ML applications with PLGA‐based formulations, elucidating trends in drug release with respect to its many features (Goren et al. 2025; Noorain et al. 2023).

Due to the prevalence of PLGA formulations and available data and assist with ML model development, many researchers have compiled reported formulations and respective drug release profiles from the literature, highlighting trends with respect to PLGA features. Zhang et al. for example, built a multilayer perceptron (MLP) neural network model “DrugNet” from 39 published formulations with corresponding release profiles (Zhang, Zhang, et al. 2025b). They defined a total of 14 parameters for this dataset, including several that were specific to the PLGA polymer (e.g., molecular weight, polydispersity index (PDI), monomer ratio). The developed MLP model outperformed classical semi‐empirical models (Korsmeyer–Peppas model and Weibull model) in fitting PLGA release profiles, achieving a substantially lower mean squared error (MSE = 13) and modestly better goodness‐of‐fit (*R* ^2^ = 0.970), demonstrating the importance PLGA features had on capturing complex release curves. This improved performance underscores the value of including PLGA‐specific features. PLGA parameters help the model capture how polymer variations influence release kinetics, while system‐based parameters such as size, loading capacity, and zeta potential reflect structural characteristics that shape release uniformity and rate. By integrating these descriptors alongside formulation and drug‐related features, the model remains grounded in the material properties that define PLGA systems while retaining enough flexibility to generalize across diverse PLGA platforms and cargos. This alignment of polymer, drug, and time inputs enables the model to learn nonlinear dependencies that traditional fixed‐form release models often fail to capture, supporting more reliable prediction across varied PLGA‐based delivery applications.

Other researchers have evaluated the influence of these polymer features to ML model predictions, such as Bannigan et al. who developed a LGBM model to predict the release of drugs from 43 unique polymeric long‐acting injectables (LAIs, microspheres and cylinders) (Bannigan et al. 2023). Of the optimized 15 features included in the model, polymer MW and LA:GA ratio was listed with polymer MW (ranked 4th) having more influence over LA/GA (ranked 12th). Furthermore, SHAP assessment revealed that polymer MW has a broadly negative correlation with its influence on ML predictions indicating higher polymer MW trends with lower release. This is expected since polymers (e.g., PLGA, polylactic acid (PLA), and polycaprolactone (PCL) included in this study) with higher MW degrade more slowly and often form denser matrices that limit drug diffusion, resulting in slower overall release kinetics (Kim et al. 2025). Although this study includes only a few different polymers within the LAI formulations, the findings highlight how ML models can capture real world relationships between molecular features of polymers and observed release behavior.

### 3.4 Drugs

Similar to the parameters that describe platforms and polymers, features that characterize the encapsulated drug are commonly included in ML models for predicting release. Polymeric systems can deliver a wide variety of therapeutic cargos ranging from small molecules to large biologics, so many physicochemical properties may be relevant. However, small molecules are most widely explored in polymeric formulations with ML (McDonald et al. 2023). Drug characteristics such as molecular weight, polar surface area, and LogP influence how molecules move through a material, and these attributes can directly affect release rates from polymeric systems (Liu and Quan 2016). Including drug‐specific parameters also helps assess model generalizability, allowing predictions to extend across different therapies. Altogether, these considerations highlight the importance of incorporating drug level features during model development.

**FIGURE 4:**
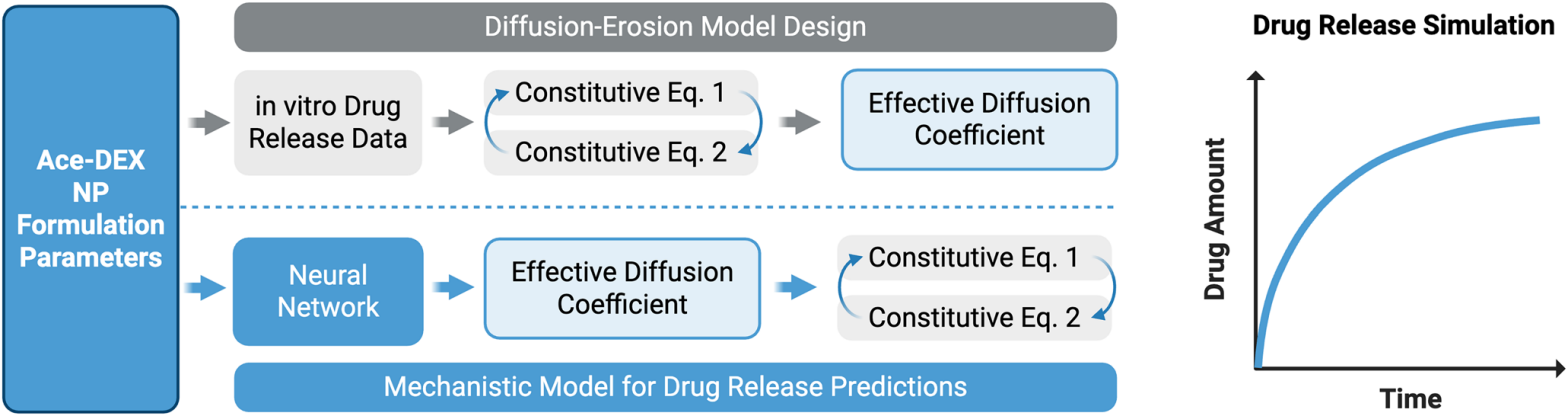
Diffusion‐erosion mechanistic model with ML for polymeric NP drug release. Flowchart of the methodology used to build (above dashed line) and develop predictability (below dashed line) the diffusion model for drug release from Ace‐DEX NPs (graph on the right). Relevant formulation parameters and corresponding drug release data were simulated using 2 constitutive equations describing spherical drug diffusion (Equation 1) and polymeric surface erosion (Equation 2). The unknown effective diffusion coefficient was solved by minimizing error from simulations in MATLAB. A predictive neural network was then employed to identify which formulation parameters most effectively captured diffusion coefficients and applied the model to future formulations.

Stiepel et al. revealed this importance of drug properties to release rates through the development of a mechanistic predictive model “diffusion‐erosion model” for characterizing drug release from Ace‐DEX nanoparticles (Stiepel et al. 2022). The mathematical model can fit drug release data across diverse formulations via least squares regression analysis and determination of the drug's effective diffusion coefficient (defined in constitutive equations, Figure 4). A Pearson correlation analysis revealed that drug polar surface area was most strongly associated with the predicted diffusion coefficients. To harness ML capabilities and predict this important parameter governing mechanistic release, a NN model was developed from data containing 22 release profiles for 4 different drugs with distinct physicochemical properties (i.e., polar surface area, LogP, MW). The diffusion‐erosion model effectively estimated drug‐specific diffusion coefficients and generally outperformed traditional release models, including zero‐order, Higuchi, and Hixson‐Crowell. The ANN accurately predicted these coefficients across formulations (Pearson's coefficient = 0.906), closely matching those derived from fitted release profiles, and reliably captured drug release profiles for two new drugs. This work shows that including detailed drug‐specific parameters allows predictive models to generalize across different Ace‐DEX formulations and more accurately capture the mechanistic relationships between drug structure and polymer behavior.

Across polymer systems, drug MW and solubility (mg/mL or LogP) are also commonly reported as having an influence on ML predictions for drug release. Sun et al. for example, developed a ML framework to investigate how drug and carrier properties influence release from PLGA micro and nanoparticles. Using a dataset compiled from prior studies, they trained several models, including GPR and an ANN, to predict drug release using four key inputs: drug solubility, MW, PLGA particle size, and release medium pH. Their analysis revealed that drug solubility was one of the strongest predictors of release, with highly soluble molecules showing faster and more complete release from PLGA matrices. Drug MW also shaped release behavior, although in a nonlinear fashion; drugs with moderate molecular weights exhibited higher release efficiencies, while very small or very large molecules released more slowly. The ML models captured these multidimensional interactions more accurately than traditional empirical approaches and guided the design of new experiments that validated the predicted trends, providing mechanistic insight into release behavior across diverse PLGA‐based systems.

Despite growing use of polymeric systems to deliver therapeutic proteins and biologics, there is still a noticeable lack of modern ML approaches designed to predict the release of these larger and more complex cargos. One early effort was reported by Szlęk et al. in 2013, who examined key features affecting macromolecule release from PLGA microsphere formulations (Szlęk et al. 2013). Their analysis compiled release data from 68 formulations in the literature and defined a large feature space that included 9 formulation parameters, 5 experimental conditions, and nearly 300 molecular descriptors for each protein, calculated using a “Marvin cxcalc plug in” tool. These descriptors captured a wide range of physicochemical, structural, and spectroscopic properties relevant to macromolecule identity. Through iterative feature reduction in an ANN model, the authors found that the best performance was achieved when the initial feature set was reduced from > 300 to 11 inputs (RMSE (%) = 15.4). Of these 11 inputs, 3 described the macromolecule itself, including a distance‐based geometry index, the isoelectric point, and the quaternary structure of the monomer/dimer. Although predictive accuracy varied across individual formulations, the study demonstrates that ML methods can efficiently identify influential properties within complex datasets and can reveal meaningful relationships between protein characteristics and release behavior from PLGA systems.

### 3.5 Complex System Features

Feature complexity can extend beyond platform structure, polymer composition, and drug properties. When compiling data from the literature, many researchers must also account for differences in assays or detection methods used across drug release studies (Zhang, Zhang, et al. 2025a). These features often include details related to the release media such as pH, temperature, volume, and sampling schedules, as well as spectroscopic measurements or analytical readouts that describe molecular interactions, stability, or structural changes during release. Including these experimental and analytical parameters can help models separate true formulation‐driven effects from variations introduced by testing methods and can strengthen ML reliability across diverse procedures. Together, these additional feature layers support more accurate and generalizable predictions of drug release.

In a study by Abdalla et al. the authors demonstrated that Raman spectral data can serve as a powerful input for ML models to predict drug release from polysaccharide coatings (Abdalla et al. 2024). They collected > 3000 observation Raman spectra for a diverse set of coatings and processed the raw data through baseline correction, normalization, and dimensionality reduction to convert the high‐dimensional spectral information into compact numerical features suitable for model training. These transformed spectral features captured chemical signatures related to glycosidic linkages, ring vibrations, and other structural elements that govern enzymatic degradation in the colon. Using these inputs, the authors trained regression models, including a random forest approach, to predict the release of 5‐amino salicylic acid under simulated colonic conditions. The model successfully predicted release profiles for new coatings not included in the training set, confirming that the processed Raman spectra encoded sufficient information about material chemistry and degradability. The study illustrates how spectral measurements can bypass manual feature engineering, allowing ML models to learn relationships between polymer structure and drug release and enabling more efficient screening of polysaccharide‐based tablets.

Galata et al. demonstrated that near infrared (NIR) spectral fingerprints can serve as powerful inputs for ML models that predict dissolution behavior of sustained release tablets (Galata et al. 2021). They collected NIR spectra from each tablet and processed the high‐dimensional data through standard spectral preprocessing steps, allowing the spectra to act as a chemical and physical signature of the formulation. These spectral features were combined with tablet compression force and polymer particle size distribution to train several ML models, including an ANN, an SVM, and an ensemble of regression trees, with the ANN providing the most accurate predictions. The use of NIR spectra allowed the models to capture subtle variation in composition, matrix structure, and excipient interactions without requiring explicit chemical descriptors, and the inclusion of polymer particle size further strengthened prediction accuracy by accounting for structural factors that influence release. Collectively, the work shows how integrating spectroscopic data with ML methods can support real time release testing and reduce the need for labor intensive dissolution studies.

## 4 Conclusions and Future Perspectives

The application of ML to predict drug release from pharmaceutical formulations has shown considerable promise, yet its advancement depends heavily on the quality and scope of available datasets (Trucillo 2022; Meyer et al. 2023). Many current models are built on limited, short‐duration release data, often under 24 h, which restricts their utility, especially for bulk‐degrading polymer systems like PLGA. For example, in the case of FDA approved Risperdal Consta, a PLGA‐based long‐acting injectable, drug release spans weeks and involves multiple kinetic phases (Yerragunta et al. 2015). However, models trained on early release profiles often capture only the initial burst phase, neglecting the sustained or secondary release behavior critical to therapeutic efficacy. This challenge highlights the need for high‐quality, standardized datasets that include longer‐term release profiles and richer metadata on formulation, processing, and environmental conditions.

To identify common trends across ML applications used to characterize polymeric drug release, we evaluated findings from recent models and highlighted patterns that may guide and streamline future work in this area (Table 6). Across the literature, input features typically describe the platform, polymer, drug, and relevant experimental conditions, along with time matched release information. Platform features with strong influence are often linked to surface morphology, including size, diameter, or surface area, and their consistent importance aligns with mechanistic expectations from conventional release theory (Woodring et al. 2025b; Owusu et al. 2025; Yuan et al. 2023). Polymer‐related attributes (although most explored across PLGA systems) such as MW, monomer ratio, and hydrophobicity show significant influence on model behavior, reinforcing the overall effect of the bulk material (Bannigan et al. 2023; Zhang, Zhang, et al. 2025a). Drug characteristics, including MW and polar surface area, commonly emerge as influential as well, which is consistent with established effects on diffusion and partitioning (Stiepel et al. 2022; Bannigan et al. 2023). Several studies have further expanded feature design by incorporating higher order measurements such as Raman or NIR spectra, demonstrating that spectral fingerprints can enhance model performance by capturing structural or compositional details that are not easily represented through traditional descriptors (Abdalla et al. 2024; Galata et al. 2021).

Despite the success of ML models and their use across polymeric formulations, many studies have indicated that a major limitation for future deployment is central to sparsity in data availability. Nevertheless, recent efforts have been made to compile and preprocess data involving diverse polymeric formulations (Pai et al. 2025). Goren et al. for example, have aimed to address these shortcomings by developing a dataset of 433 unique PLGA nanoparticles with 18 features and encapsulating a total of 65 small molecule drugs (Goren et al. 2025). Growing these data sets will strengthen robustness for ML applications in the future. Furthermore, integration of experimental and simulated data is emerging as a powerful strategy to augment sparse datasets (Cheung et al. 2024). Simulations based on mechanistic understanding of degradation and diffusion processes can complement experimental release profiles, helping to fill gaps and improve generalizability.

Even with such advancements, several critical limitations remain, including the lack of standardized data reporting, inconsistent experimental conditions, and limited data harmonization across studies, all of which hinder model transferability and reproducibility (Brancato et al. 2024; Li et al. 2025). Addressing these challenges will require community‐wide efforts toward harmonized data formats, shared descriptors for polymer and drug features, and consistent reporting of experimental metadata. In parallel, future ML models should integrate uncertainty quantification frameworks to assess prediction confidence and reliability, particularly when extrapolating beyond the training domain (Shi et al. 2025). Additionally, the widespread use of black‐box ML models presents interpretability challenges that may limit adoption in formulation design and regulatory contexts (Mohamed et al. 2025). Incorporating interpretable ML methods or hybrid approaches that couple mechanistic models with data‐driven learning could improve transparency, build trust, and enhance the translational impact of ML‐enabled drug release modeling.

Recent innovations in transfer learning and generative models offer exciting opportunities to overcome data scarcity that might occur in some studies. Transfer learning allows models trained on one formulation type to be fine‐tuned for another, reducing the data burden for novel formulations (Zhao et al. 2024). Meanwhile, generative models such as variational autoencoders (VAEs) and generative adversarial networks (GANs) can propose new formulations with predicted release characteristics, accelerating design cycles (Alsafadi and Wu 2023). Additionally, physics‐informed ML that uses prior knowledge (e.g., mechanistic equations) to establish model architecture, which can offer a hybrid approach that leverages both data and constitutive equations, particularly in systems where physical constraints (e.g., diffusion, erosion, swelling) dominate behavior (Malashin et al. 2025).

As ML continues to be integrated into pharmaceutical research, ethical considerations must also be addressed (Hanna et al. 2025; Arabi 2021). These include ensuring transparency and interpretability of model predictions, preventing algorithmic bias introduced by unrepresentative or poor‐quality data, and safeguarding proprietary or sensitive experimental data. Ethical deployment of ML models will require careful attention to data privacy, fairness, and regulatory compliance, particularly as such models begin to influence pre‐clinical and clinical decision‐making.

Looking forward, one of the most impactful frontiers lies in bridging the gap between in vitro release predictions and in vivo pharmacokinetics (PK). Most ML models focus solely on in vitro behavior, but future models will increasingly integrate drug release kinetics with physiological parameters (e.g., enzymatic activity, tissue perfusion) to predict systemic exposure (Cavasotto and Scardino 2022). Such in vitro–in vivo correlation (IVIVC) models, empowered by ML, could significantly improve formulation design and reduce reliance on animal studies (Lu et al. 2011; Peloso et al. 2025).

In summary, while ML has proven effective for modeling drug release, its future success hinges on expanded datasets, better integration with simulations, and adoption of emerging techniques like transfer learning and physics‐informed modeling. Progress in these areas will be critical to enabling predictive, reliable, and clinically relevant drug delivery design.

## Author Contributions

**Ryan N. Woodring:** conceptualization (equal), writing – original draft (equal), writing – review and editing (equal). **Kristy M. Ainslie:** conceptualization (equal), writing – original draft (equal), writing – review and editing (equal).

## Funding

This work was supported by the National Institute of Health (NIH R01CA257009) and Pharmalliance Research Clusters for Doctoral Training (PARCDT) sponsorship for graduate research.

## Conflicts of Interest

The authors declare no conflicts of interest.

## Related WIREs Articles

Combining machine‐learning and molecular‐modeling methods for drug‐target affinity predictions.

## Supporting information


**Data S1:** wnan70057‐sup‐0001‐Supinfo.docx.

## Data Availability

This review article does not contain any original data. All information discussed is derived from previously published sources, which are appropriately cited throughout the manuscript.
