# Supplementary material for: Modeling Polymeric Drug Release: The Emerging Role of Machine Learning
Source: Wiley Interdiscip Rev Nanomed Nanobiotechnol. 2026 Mar 2;18(2):e70057. doi: 10.1002/wnan.70057 (PMC12953059; doi:10.1002/wnan.70057)
Supplement: Supplementary file 1 — Data S1: wnan70057‐sup‐0001‐Supinfo.docx. [file WNAN-18-e70057-s001.docx]

**SUPPLEMENTAL**

**Supplemental Table S1:** Common equations defined for mechanistic and empirical drug release models

| Model | General Equation | Definition(s) |
| --- | --- | --- |
| Fick’s First Law | $J=-D\frac{\partial C}{\partial x}$ | The flux (J) of a drug is proportional to the diffusion coefficient (D) and concentration gradient ($\frac{\boldsymbol{\partial C}}{\boldsymbol{\partial x}}$) |
| Fick’s Second Law | $\frac{\partial C}{\partial t}=D\frac{\partial^{2}C}{\partial x^{2}}$ | The change in drug concentration ($\frac{\boldsymbol{\partial C}}{\boldsymbol{\partial t}})$is proportional to the second spatial derivative of the concentration profile ($\frac{\boldsymbol{\partial}^{\mathbf{2}}\mathbf{C}}{\boldsymbol{\partial}\mathbf{x}^{\mathbf{2}}})$ |
| Higuchi | $Q=k_{H}\cdot t^{\frac{1}{2}}$ | The amount of drug released per unit area (Q) is governed by Fickian diffusion (k_H_, Higuchi release constant) and increases with square root of time |
| Peppas-Sahlin | $\frac{M_{t}}{M_{\infty}}=k_{1}t^{m}+k_{2}t^{2m}$ | The drug fraction released ($\frac{\mathbf{M}_{\mathbf{t}}}{\mathbf{M}_{\infty}})$ is the sum of Fickian diffusion ($\mathbf{k}_{\mathbf{1}}\mathbf{t}^{\mathbf{m}}$) and polymer relaxation/swelling ($\mathbf{k}_{\mathbf{2}}\mathbf{t}^{\mathbf{2m}}$) where m is the diffusional exponent related to the respective mechanisms |
| Hixson-Crowell | ${W_{0}}^{1/3}-{W_{t}}^{1/3}=k\cdot t$ | The relationship between the cubed root amount of drug remaining (W_t_) and the initial amount of loaded drug (W_0_) is proportional to the rate of matrix degradation (k, rate constant) |
| Göpferich | $\frac{M_{t}}{M_{\infty}}=1-(1-kt)^{n}$ | Drug release relating polymer degradation rate (k) and a release exponent (n) representing matrix geometry and erosion |
| Korsmeyer-Peppas | $\frac{M_{t}}{M_{\infty}}=kt^{n}$ | Drug release relating kinetic constant for structural geometry (k), time, and mechanistic release exponent (n) |
| Zero-Order | $M_{t}=M_{0}+k_{0}t$ | Drug release (M_t_) at time t is linearly proportional to the initial amount of drug (M_0_) plus the release constant (k_0_) times time (t). |
| First-Order | $\frac{\partial M}{\partial t}=-k_{1}M$ | The rate of drug release ($\frac{\boldsymbol{\partial M}}{\boldsymbol{\partial t}}$) is proportional to the amount of drug remaining (M) at time t. k_1_ is the release rate constant. |
| Weibull | $\frac{M_{t}}{M_{\infty}}=1-exp{[-(\frac{t-T}{\alpha})}^{\beta}]$ | Drug release relating time (t), lag time prior to release (T), release time scale ($\boldsymbol{\alpha}$), and kinetic shape parameter ($\boldsymbol{\beta}$). |
| Logistic | $\frac{M_{t}}{M_{\infty}}=\frac{1}{1+e^{-k(t-t_{0})}}$ | Sigmoidal drug release with a rate constant for curve steepness (k) and time when release inflects (t_0_) |
| Polynomial | $M_{t}=a_{0}+a_{1}t+a_{2}t^{2}+\ldots+a_{n}t^{n}$ | Drug release relating fitted coefficients (a_0-n_) to time and the polynomial degree (n) |
